# 2-Cyano­anilinium tetra­fluoro­borate

**DOI:** 10.1107/S1600536809032863

**Published:** 2009-09-09

**Authors:** Yi Zhang

**Affiliations:** a Ordered Matter Science Research Center, College of Chemistry and Chemical Engineering, Southeast University, Nanjing 210096, People’s Republic of China, and Department of Physics, Southeast University, Nanjing 210096, People’s Republic of China

## Abstract

In the title compound, C_7_H_7_N_2_^+^·BF_4_^−^, the non-H atoms of the cation are almost coplanar (r.m.s. deviation = 0.035 Å). The cations and anions are connected by inter­molecular N—H⋯F and N—H⋯N hydrogen bonds, forming a two-dimensional network parallel to (10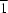).

## Related literature

For the application of metal-organic coordination compounds, see: Fu *et al.* (2007 ▶); Chen *et al.* (2000 ▶); Fu & Xiong (2008 ▶); Xiong *et al.* (1999 ▶); Xie *et al.* (2003 ▶); Zhang *et al.* (2001 ▶). For general background to nitrile derivatives, see: Fu *et al.* (2008 ▶); Wang *et al.* (2002 ▶).
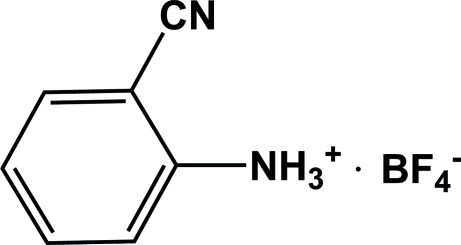


## Experimental

### 

#### Crystal data

C_7_H_7_N_2_^+^·BF_4_^−^
*M* *_r_* = 205.96
Monoclinic, 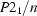

*a* = 10.971 (2) Å
*b* = 7.3565 (15) Å
*c* = 11.022 (2) Å
β = 103.11 (3)°
*V* = 866.4 (3) Å^3^
*Z* = 4
Mo *K*α radiation
μ = 0.16 mm^−1^
*T* = 298 K
0.30 × 0.25 × 0.20 mm

#### Data collection

Rigaku Mercury2 diffractometer
Absorption correction: multi-scan (*CrystalClear*; Rigaku, 2005 ▶) *T*_min_ = 0.96, *T*_max_ = 1.00 (expected range = 0.931–0.969)
8625 measured reflections
1978 independent reflections
1417 reflections with *I* > 2σ(*I*)
*R*_int_ = 0.041

#### Refinement

*R*[*F* ^2^ > 2σ(*F* ^2^)] = 0.056
*wR*(*F* ^2^) = 0.141
*S* = 1.08
1978 reflections
128 parameters
H-atom parameters constrained
Δρ_max_ = 0.32 e Å^−3^
Δρ_min_ = −0.32 e Å^−3^

### 

Data collection: *CrystalClear* (Rigaku, 2005 ▶); cell refinement: *CrystalClear*; data reduction: *CrystalClear*; program(s) used to solve structure: *SHELXS97* (Sheldrick, 2008 ▶); program(s) used to refine structure: *SHELXL97* (Sheldrick, 2008 ▶); molecular graphics: *SHELXTL* (Sheldrick, 2008 ▶); software used to prepare material for publication: *SHELXTL*.

## Supplementary Material

Crystal structure: contains datablocks I, global. DOI: 10.1107/S1600536809032863/ci2881sup1.cif

Structure factors: contains datablocks I. DOI: 10.1107/S1600536809032863/ci2881Isup2.hkl

Additional supplementary materials:  crystallographic information; 3D view; checkCIF report

## Figures and Tables

**Table 1:** Hydrogen-bond geometry (Å, °)

*D*—H⋯*A*	*D*—H	H⋯*A*	*D*⋯*A*	*D*—H⋯*A*
N1—H1*A*⋯N2^i^	0.89	2.54	3.179 (3)	130
N1—H1*A*⋯F4^ii^	0.89	2.12	2.896 (2)	146
N1—H1*B*⋯F4^iii^	0.89	2.21	2.993 (2)	147
N1—H1*C*⋯F3	0.89	1.95	2.799 (2)	160

Symmetry codes: (i) 
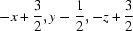
; (ii) 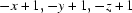; (iii) 
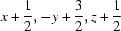
.

**Fig. 1.**
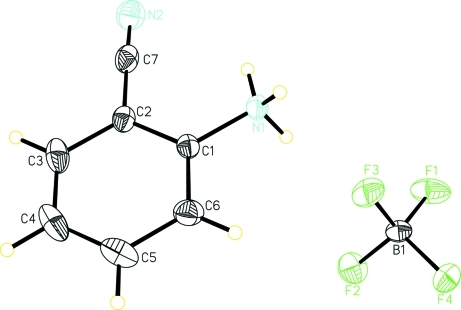
A view of the title compound with the atomic numbering scheme. Displacement ellipsoids are drawn at the 30% probability level.

**Fig. 2.**
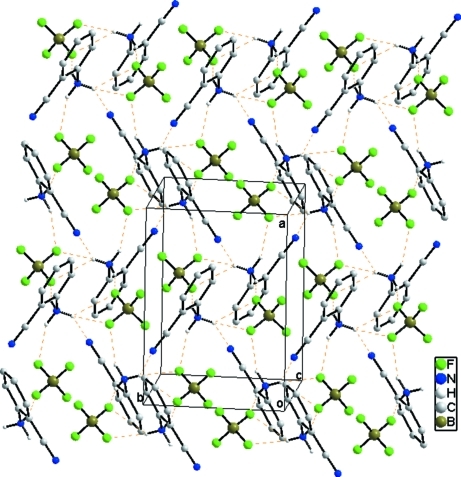
The crystal packing of the title compound, viewed along the c axis. Hydrogen bonds are shown as dashed lines. C-bound H atoms have been omitted for clarity

## supplementary crystallographic information

### Comment

The construction of metal-organic coordination compounds has attracted much attention owing to potential functions, such as permittivity, fluorescence, magnetism and optical properties (Fu *et al.*, 2007; Chen *et al.*, 2000; Fu & Xiong (2008); Xie *et al.*, 2003; Zhang *et al.*,2001; Xiong *et al.*, 1999). Nitrile derivatives are a class of excellent ligands for the construction of novel metal-organic frameworks. (Wang *et al.* 2002; Fu *et al.*, 2008). We report here the crystal structure of the title compound, 2-cyanobenzenaminium tetrafluoroborate.

In the 2-cyanobenzenaminium cation (Fig.1), the nitrile group and the benzene ring are almost coplanar. The nitrile group C7≡N2 bond length of 1.150 (3) Å is within the normal range.

In the crystal structure, all the amine group H atoms are involved in N—H···F hydrogen bonds (Table 1) with F atoms of the BF_4_^-^ anion. These hydrogen bonds along with N—H···N hydrogen bonds link the ionic units into a two-dimensional network (Fig. 2) parallel to the (101).

### Experimental

The commercial 2-aminobenzonitrile (3 mmol, 0.55 g) and HBF_4_ (0.5 ml) were dissolved in ethanol (20 ml). Colourless block-shaped crystals of the title compound suitable for X-ray analysis were obtained by slow evaporation at room temperature.

### Refinement

All H atoms attached to C and N atoms were positioned geometrically and treated as riding, with C-H = 0.93 Å, N-H = 0.89 Å and *U*_iso_(H) = 1.2U_eq_(C) and *U*_iso_(H) = 1.5U_eq_(N). A rotating-group model was used for the -NH_3_ group.

### Crystal data

**Table:**

C_7_H_7_N_2_^+^·BF_4_^−^	*F*(000) = 416
*M**_r_* = 205.96	*D*_x_ = 1.579 Mg m^−^^3^
Monoclinic, *P*2_1_/*n*	Mo *K*α radiation, λ = 0.71073 Å
Hall symbol: -P 2yn	Cell parameters from 1417 reflections
*a* = 10.971 (2) Å	θ = 3.4–27.5°
*b* = 7.3565 (15) Å	µ = 0.16 mm^−^^1^
*c* = 11.022 (2) Å	*T* = 298 K
β = 103.11 (3)°	Block, colourless
*V* = 866.4 (3) Å^3^	0.30 × 0.25 × 0.20 mm
*Z* = 4	

### Data collection

**Table:**

Rigaku Mercury2 diffractometer	1978 independent reflections
Radiation source: fine-focus sealed tube	1417 reflections with *I* > 2σ(*I*)
graphite	*R*_int_ = 0.041
Detector resolution: 13.6612 pixels mm^-1^	θ_max_ = 27.5°, θ_min_ = 3.4°
CCD profile fitting scans	*h* = −14→14
Absorption correction: multi-scan (*CrystalClear*; Rigaku, 2005)	*k* = −9→9
*T*_min_ = 0.96, *T*_max_ = 1.00	*l* = −14→14
8625 measured reflections	

### Refinement

**Table:**

Refinement on *F*^2^	Primary atom site location: structure-invariant direct methods
Least-squares matrix: full	Secondary atom site location: difference Fourier map
*R*[*F*^2^ > 2σ(*F*^2^)] = 0.056	Hydrogen site location: inferred from neighbouring sites
*wR*(*F*^2^) = 0.141	H-atom parameters constrained
*S* = 1.08	*w* = 1/[σ^2^(*F*_o_^2^) + (0.0571*P*)^2^ + 0.4139*P*] where *P* = (*F*_o_^2^ + 2*F*_c_^2^)/3
1978 reflections	(Δ/σ)_max_ = 0.001
128 parameters	Δρ_max_ = 0.32 e Å^−^^3^
0 restraints	Δρ_min_ = −0.32 e Å^−^^3^

### Special details

**Table:**

Geometry. All e.s.d.'s (except the e.s.d. in the dihedral angle between two l.s. planes) are estimated using the full covariance matrix. The cell e.s.d.'s are taken into account individually in the estimation of e.s.d.'s in distances, angles and torsion angles; correlations between e.s.d.'s in cell parameters are only used when they are defined by crystal symmetry. An approximate (isotropic) treatment of cell e.s.d.'s is used for estimating e.s.d.'s involving l.s. planes.
Refinement. Refinement of *F*^2^ against ALL reflections. The weighted *R*-factor *wR* and goodness of fit *S* are based on *F*^2^, conventional *R*-factors *R* are based on *F*, with *F* set to zero for negative *F*^2^. The threshold expression of *F*^2^ > σ(*F*^2^) is used only for calculating *R*-factors(gt) *etc*. and is not relevant to the choice of reflections for refinement. *R*-factors based on *F*^2^ are statistically about twice as large as those based on *F*, and *R*- factors based on ALL data will be even larger.

### Fractional atomic coordinates and isotropic or equivalent isotropic displacement parameters (Å^2^)

**Table:**

	*x*	*y*	*z*	*U*_iso_*/*U*_eq_	
F4	0.36113 (13)	0.72246 (19)	0.25229 (13)	0.0500 (4)	
F3	0.48625 (14)	0.7353 (2)	0.44366 (13)	0.0575 (4)	
N1	0.59296 (16)	0.6600 (3)	0.69414 (15)	0.0337 (4)	
H1A	0.6182	0.5482	0.6812	0.051*	
H1B	0.6589	0.7275	0.7293	0.051*	
H1C	0.5548	0.7094	0.6218	0.051*	
F2	0.34337 (15)	0.9602 (2)	0.37300 (16)	0.0625 (5)	
C1	0.50557 (18)	0.6518 (3)	0.77689 (18)	0.0302 (5)	
F1	0.51445 (14)	0.9330 (3)	0.29556 (18)	0.0737 (6)	
C2	0.5337 (2)	0.7425 (3)	0.89064 (19)	0.0348 (5)	
C6	0.3969 (2)	0.5547 (3)	0.7402 (2)	0.0437 (6)	
H6	0.3792	0.4939	0.6643	0.052*	
N2	0.7317 (2)	0.9429 (3)	0.9488 (2)	0.0586 (6)	
C7	0.6444 (2)	0.8517 (3)	0.9248 (2)	0.0428 (6)	
B1	0.4282 (2)	0.8402 (3)	0.3425 (2)	0.0359 (6)	
C4	0.3405 (3)	0.6365 (4)	0.9311 (3)	0.0580 (8)	
H4	0.2840	0.6313	0.9824	0.070*	
C3	0.4507 (2)	0.7323 (3)	0.9687 (2)	0.0483 (6)	
H3	0.4693	0.7897	1.0458	0.058*	
C5	0.3138 (2)	0.5485 (4)	0.8180 (3)	0.0570 (7)	
H5	0.2393	0.4842	0.7934	0.068*	

### Atomic displacement parameters (Å^2^)

**Table:**

	*U*^11^	*U*^22^	*U*^33^	*U*^12^	*U*^13^	*U*^23^
F4	0.0566 (9)	0.0453 (8)	0.0442 (8)	−0.0023 (6)	0.0037 (7)	−0.0056 (6)
F3	0.0587 (9)	0.0613 (10)	0.0455 (8)	0.0069 (7)	−0.0029 (7)	0.0162 (7)
N1	0.0376 (10)	0.0351 (9)	0.0291 (9)	0.0028 (8)	0.0092 (7)	−0.0004 (7)
F2	0.0658 (9)	0.0467 (9)	0.0762 (11)	0.0167 (7)	0.0186 (8)	−0.0097 (8)
C1	0.0324 (10)	0.0288 (10)	0.0303 (10)	0.0072 (8)	0.0091 (8)	0.0036 (8)
F1	0.0435 (8)	0.0830 (12)	0.0922 (13)	−0.0162 (8)	0.0107 (8)	0.0342 (10)
C2	0.0420 (11)	0.0316 (11)	0.0311 (11)	0.0065 (9)	0.0091 (9)	0.0001 (9)
C6	0.0356 (12)	0.0447 (14)	0.0491 (14)	0.0034 (10)	0.0058 (10)	−0.0046 (11)
N2	0.0597 (14)	0.0587 (14)	0.0558 (14)	−0.0010 (12)	0.0095 (11)	−0.0178 (11)
C7	0.0542 (15)	0.0392 (13)	0.0347 (12)	0.0088 (12)	0.0094 (11)	−0.0075 (10)
B1	0.0324 (12)	0.0315 (12)	0.0412 (13)	0.0004 (10)	0.0030 (10)	0.0053 (11)
C4	0.0609 (17)	0.0541 (17)	0.0729 (19)	0.0135 (14)	0.0445 (15)	0.0118 (14)
C3	0.0670 (16)	0.0430 (13)	0.0422 (13)	0.0147 (12)	0.0277 (12)	0.0018 (10)
C5	0.0375 (13)	0.0529 (16)	0.085 (2)	0.0011 (12)	0.0223 (13)	0.0022 (14)

### Geometric parameters (Å, °)

**Table:**

F4—B1	1.396 (3)	C2—C3	1.389 (3)
F3—B1	1.387 (3)	C2—C7	1.434 (3)
N1—C1	1.467 (2)	C6—C5	1.387 (4)
N1—H1A	0.89	C6—H6	0.93
N1—H1B	0.89	N2—C7	1.150 (3)
N1—H1C	0.89	C4—C5	1.376 (4)
F2—B1	1.379 (3)	C4—C3	1.379 (4)
C1—C6	1.370 (3)	C4—H4	0.93
C1—C2	1.392 (3)	C3—H3	0.93
F1—B1	1.361 (3)	C5—H5	0.93
			
C1—N1—H1A	109.5	F1—B1—F2	109.7 (2)
C1—N1—H1B	109.5	F1—B1—F3	110.68 (19)
H1A—N1—H1B	109.5	F2—B1—F3	111.9 (2)
C1—N1—H1C	109.5	F1—B1—F4	109.9 (2)
H1A—N1—H1C	109.5	F2—B1—F4	107.16 (18)
H1B—N1—H1C	109.5	F3—B1—F4	107.42 (19)
C6—C1—C2	121.1 (2)	C5—C4—C3	120.3 (2)
C6—C1—N1	119.06 (19)	C5—C4—H4	119.9
C2—C1—N1	119.87 (19)	C3—C4—H4	119.9
C3—C2—C1	119.3 (2)	C4—C3—C2	119.7 (2)
C3—C2—C7	120.2 (2)	C4—C3—H3	120.2
C1—C2—C7	120.42 (19)	C2—C3—H3	120.2
C1—C6—C5	119.0 (2)	C4—C5—C6	120.6 (2)
C1—C6—H6	120.5	C4—C5—H5	119.7
C5—C6—H6	120.5	C6—C5—H5	119.7
N2—C7—C2	177.7 (3)		
			
C6—C1—C2—C3	0.8 (3)	C5—C4—C3—C2	1.2 (4)
N1—C1—C2—C3	−179.40 (19)	C1—C2—C3—C4	−1.6 (3)
C6—C1—C2—C7	−176.6 (2)	C7—C2—C3—C4	175.8 (2)
N1—C1—C2—C7	3.2 (3)	C3—C4—C5—C6	0.0 (4)
C2—C1—C6—C5	0.4 (3)	C1—C6—C5—C4	−0.8 (4)
N1—C1—C6—C5	−179.4 (2)		

### Hydrogen-bond geometry (Å, °)

**Table:**

*D*—H···*A*	*D*—H	H···*A*	*D*···*A*	*D*—H···*A*
N1—H1A···N2^i^	0.89	2.54	3.179 (3)	130
N1—H1A···F4^ii^	0.89	2.12	2.896 (2)	146
N1—H1B···F4^iii^	0.89	2.21	2.993 (2)	147
N1—H1C···F3	0.89	1.95	2.799 (2)	160

Symmetry codes: (i) −*x*+3/2, *y*−1/2, −*z*+3/2; (ii) −*x*+1, −*y*+1, −*z*+1; (iii) *x*+1/2, −*y*+3/2, *z*+1/2.
